# Gut microbiota and acne: A Mendelian randomization study

**DOI:** 10.1111/srt.13473

**Published:** 2023-09-18

**Authors:** Qiurui Cao, Jinyan Guo, Shuangqing Chang, Zhifang Huang, Qinghua Luo

**Affiliations:** ^1^ Department of Anorectal Surgery Jiangmen Wuyi Hospital of Traditional Chinese Medicine Jiangmen China; ^2^ Clinical Medical College Jiangxi University of Traditional Chinese Medicine Nanchang China

**Keywords:** acne, causal effect, gut microbiota, GWAS, Mendelian randomization

## Abstract

**Background:**

Prior observational studies have identified a relationship between the composition of gut microbiota and the onset of acne. To ascertain the causal relationship underlying this association, we adopted the Mendelian randomization (MR) method, which offers a powerful approach to causal inference.

**Methods:**

Summary statistics on gut microbiota and acne were obtained from the MiBioGen and FinnGen consortium, respectively. The causal relationship was assessed using multiple methods in a two‐sample framework, including MR Egger, weighted median, inverse variance weighted (IVW), and weighted mode. Furthermore, the heterogeneity and horizontal pleiotropy analyses were conducted, along with the leave‐one‐out method.

**Results:**

The IVW estimation indicated that *Allisonella* (odds ratio [OR] = 1.42, 95% confidence interval [CI] = 1.18–1.70, *p* = 0.0002) and *Bacteroides* (OR = 2.25, 95% CI = 1.48–3.42, *p* = 0.0001) have adverse effects on acne. By contrast, *Ruminococcus torques* group (OR = 0.41, 95% CI = 0.25–0.65, *p* = 0.0002) showed a beneficial effect on acne. In addition, *Candidatus soleaferrea* (OR = 0.75, 95% CI = 0.60–0.95, *p* = 0.0149), *Eubacterium coprostanoligenes* group (OR = 0.67, 95% CI = 0.47–0.95, *p* = 0.0230), *Fusicatenibacter* (OR = 0.71, 95% CI = 0.52–0.97, *p* = 0.02897), and *Lactobacillus* (OR = 0.72, 95% CI = 0.58–0.90, *p* = 0.0046) showed suggestive associations with acne.

**Conclusion:**

The present investigation suggests a causal effect of gut microbiota on acne.

## 1 INTRODUCTION

Acne, a prevalent and chronic inflammatory skin disease, predominantly impacts young individuals. It ranks as the eighth most common disease globally, with a morbidity rate of 9.4%.^1^, ^2^ The condition involves the formation of lesions in the sebaceous glands of the hair follicles and is characterized by four factors: excessive sebum production, increased skin keratinization, accumulation of *Propionibacterium* acnes, and ensuing inflammatory response.^3^, ^4^ Acne has four clinical features: open acne, papules, pustules, and nodules.^5^ Extensive research indicates that the genetic architecture of acne is complex, involving multiple susceptibility loci. This reflects the multifactorial nature of acne pathogenesis, which involves innate immune system function, variable inflammation production, modified lipogenesis, and androgen overproduction. These factors warrant careful consideration in the understanding and management of acne.^6^, ^7^ Acne can lead to a multitude of adverse effects. Physically, it can cause discomfort, negatively impact the aesthetic appearance of the skin, and even result in permanent scarring. Psychologically, it can undermine the self‐confidence of patients, induce anxiety and embarrassment, and substantially impair social skills.^8^, ^9^

The human gut possesses a diverse microbial community that plays a vital function in the pathogenesis of acne.^10^ According to a recent study, there was a reduction in the abundance of *actinomycetes, bifidobacterium, butyricococcus*, fecal *bacillus*, and *Lactobacillus* in the intestinal microbiota of individuals with acne. Conversely, an increase in abundance of proteus was found in this population.^11^ Another study including 31 patients with acne found similar results.^12^ However, some studies reported inconsistent results. For instance, Deng et al. reported a reduction in bacillus abundance in individuals with acne,^13^ whereas Volkova et al. observed an increase in its abundance.^14^ Many studies investigating the relationship between gut microbiota and acne have utilized a case‐control study design, which makes it challenging to establish a clear causal relationship between the two. Observational studies are prone to the influence of various confounding factors, including but not limited to environmental factors, age, dietary habits, and lifestyle, which may have an impact on the results.^15^ Undoubtedly, in an observational study, it is challenging to control for all potential confounding factors. Hence, there is a dire need to apply alternative methods to infer a causal relationship between gut microbiota and acne.

Mendelian randomization (MR) is a commonly employed method to study possible causal relationships between exposures and outcomes,^16^ as it is free from confounding and reverse causation. This approach is based on Mendel's law, which involves the “random assignment of parental alleles to offspring” and mimics the randomization process of randomized controlled trials (RCTs). MR has proven to be a valuable tool in exploring causal association between complex traits or diseases and phenotypes. Recently, it has been applied to study the causal link between gut microbiota and various diseases, including mental disorders,^17^ cardiovascular diseases,^18^ and cancer.^19^ In this particular study, we employed a two‐sample MR analysis to assess the potential relationship between gut microbiota and acne, using genome‐wide association study (GWAS) summary statistics from the MiBioGen consortium and FinnGen study.

## 2 MATERIALS AND METHODS

### 2.1 Data sources

The MiBioGen consortium's genome‐wide meta‐analysis provides a comprehensive collection of genetic variations related to gut microbiota. This database is presently considered the most extensive compilation of its kind.^20^ The study comprises 24 cohorts and involves 18,340 individuals, among which microbial composition was analyzed for variable regions V3, V4‐V1, and V2‐V16 of the 16S rRNA gene as study targets using direct taxonomy for classification. A microbiota quantitative trait loci (mbQTL) localization analysis was performed to find host genetic variants and localize them to genetic loci associated with bacterial taxon abundance in the gut microbiota. This study includes five hierarchies, namely genus, family, order, class, and phylum, with genus being the lowest level. A total of 131 attribute units, of which 12 were unknown, were defined as exceeding 1% of abundance. Ultimately, 119 known units at the genus level were examined in this study. The latest published summary‐level statistics on acne were obtained in FinnGen GWAS results (https://r8.finngen.fi/), including 2,313 patients with acne and 328,747 controls.^21^ The corresponding phenotype code was “L12_ACNE.” ICD‐10 (International Classification of Diseases) is used to define acne.

All of the data used in this study were sourced from publicly available publications, and therefore, ethical approval or patient consent was not required for the analysis.

### 2.2 Instrumental variable (IV)

To screen for instrumental variables, the following criteria were applied: (1) a relaxed significance threshold of *p* < 1.0 × 10^−5^ was set to identify single nucleotide polymorphisms (SNPs) associated with the exposure, considering the limited number of genome‐wide significant SNPs for gut microbiota^22^; (2) an r^2^ threshold of 0.001 and a clump window size of 10,000 kb were set to exclude linkage disequilibrium (LD) interference; (3) SNPs with minor allele frequency (MAF) ≤0.01 were removed; (4) Palindromic SNPs were removed to avoid bias due to inconsistent strand orientation.

### 2.3 Statistical analysis

To obtain more comprehensive and accurate results, several MR analysis approaches were employed, including inverse variance weighted (IVW), MR Egger, weighted median (WM), and weighted mode. The IVW method, the most commonly used MR method, assigns weights based on the inverse variance of each instrumental variable and assumes the validity of all instruments.^23^ The MR‐Egger method uses a form of weighted linear regression analysis and is more robust to invalid instrumental variables, although it may be less statistically precise and more susceptible to outlying genetic variation.^24^ Moreover, WM takes into account the considerable variation in estimation accuracy and generally uses inverse weights of variance for each genetic variant similar to the IVW method. This method is more reliable in violating causal effects.^25^ The weighted model technique is still applicable even if other instrumental variables fail to meet the criteria for causal inference, as long as the majority of instruments have comparable causal estimates.^26^ OR and 95% CI were used to evaluate the degree of effect.

Pleiotropy was evaluated using the MR‐Egger regression intercept. In addition, the MR‐PRESSO test can help to determine the presence of pleiotropy, identify outlying SNPs, and correct them.^27^ Heterogeneity in the IVW and MR‐Egger methods was assessed using Cochran's Q statistic. A *p* value <0.05 indicates the presence of heterogeneity, while a higher *p* value indicates otherwise. Sensitivity analysis was performed using the leave‐one‐out method. In this method, each SNP was successively removed, and the combined effect of the remaining SNPs was calculated to determine whether a genetic variant has a substantial influence on the effect.^28^

The F statistics, which effectively assesses the strength of instrumental variables, is calculated as follows: *F* = *R* ^2^(*n*‐*k*‐1)/*k*(1‐*R* ^2^), where *R* ^2^, *N*, and *k* represent the proportion of variance, sample size, and number of instruments in the exposure explained by genetic variation, respectively.^29^ When the F‐statistic is higher than 10, it is suggestive of the absence of weak instrument bias.^23^

A false discovery rate (FDR) correction was carried out to account for multiple comparisons, with an FDR q‐value of less than 0.1 defined as significance.^30^ A suggestive correlation between gut microbiota and acne was deemed to exist if *p* was less than 0.05 and *q* was greater than or equal to 0.1.

The analyses were accomplished using R version 4.2.2, utilizing the “TwoSampleMR”^31^ and “MRPRESSO”^24^ software packages.

## 3 RESULTS

Based on the selection criteria for instrumental variables (refer to Table S1), 1531 SNPs were utilized to investigate 119 bacterial genera and acne. Additional information regarding the instrumental variables for the seven microflora‐acne associations can be found in Table S2.

**FIGURE 1:**
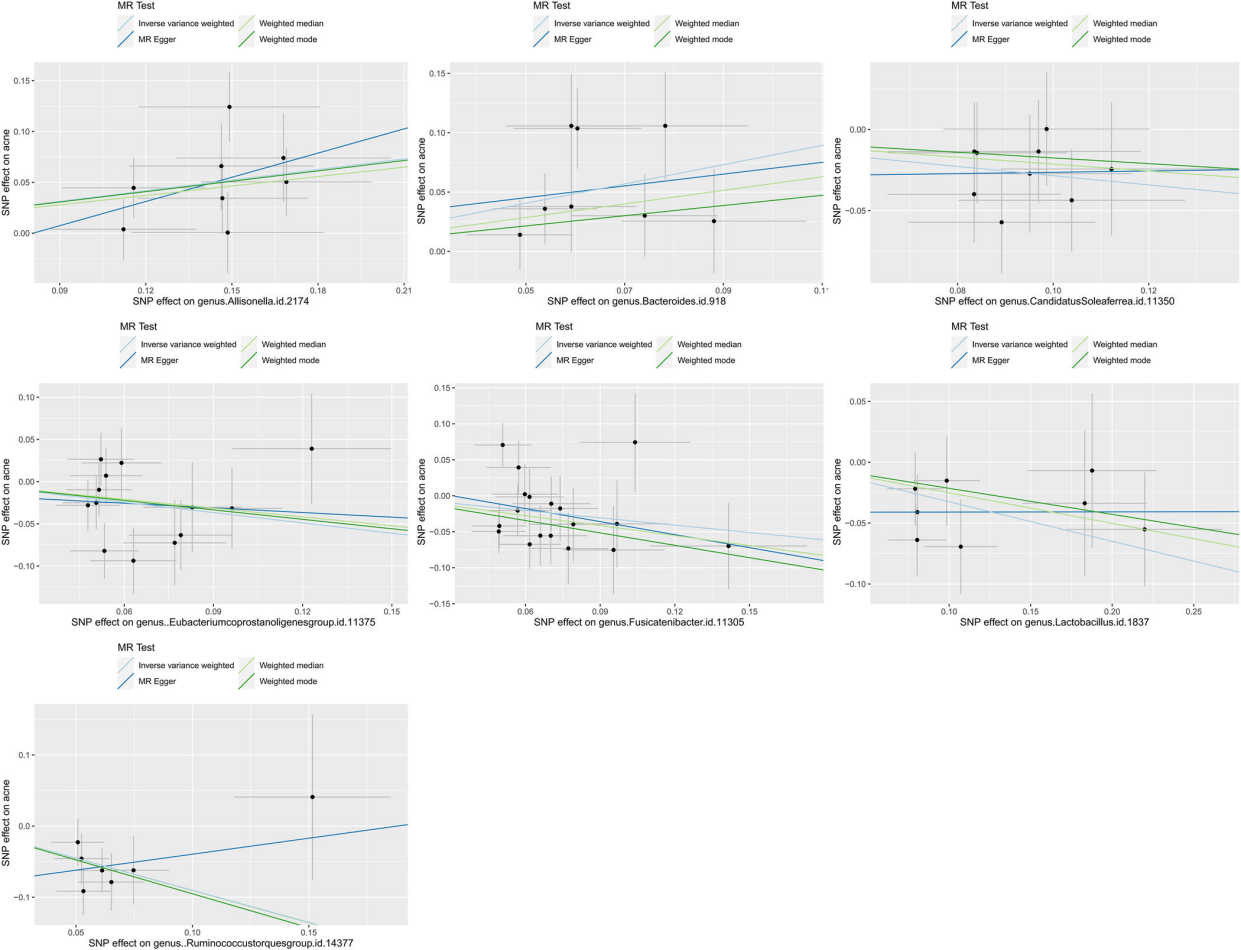
Scatter plot of the causal relationship between gut microbiome and acne.

Table 1 and Figure 1 present the findings, indicating that seven bacterial genera, namely *Allisonella, Bacteroides, Candidatus Soleaferrea, Eubacterium coprostanoligenes* group, *Fusicatenibacter, Lactobacillus*, and *Ruminococcus torques* group that have p‐values below 0.05 in the IVW analysis. The IVW analysis revealed that Allisonella (OR = 1.42, 95%, CI = 1.18–1.70, *p* = 0.0002, *q* = 0.0095) and *Bacteroides* (OR = 2.25, 95% CI = 1.48*–*3.42, *p* = 0.0001, *q* = 0.0095) had a deleterious effect on acne, while *Ruminococcus torques* group (IVW OR = 0.41, 95% CI = 0.25–0.65, *p* = 0.0002, *q* = 0.0095) showed a protective role against acne. After FDR correction, the study found that *Candidatus soleaferrea* (IVW OR = 0.75, 95% CI = 0.60–0.95, *p* = 0.0149, *q* = 0.3896), *Eubacterium coprostanoligenes* group (IVW OR = 0.67, 95% CI = 0.47–0.95, *p* = 0.0230, *q* = 0.5013), *Fusicatenibacter* (IVW OR = 0.71, 95% CI = 0.52–0.97, *p* = 0.02897, *q* = 0.5422), *Lactobacillus* (IVW OR = 0.72, 95% CI = 0.58–0.90, *p* = 0.0046, *q* = 0.1491) showed suggestive, negative associations with acne.

**TABLE 1:** MR estimates for the association between gut microbiota and acne.

Bacterial taxa (exposure)	nSNPs	Method	OR (95% CI)	*p*‐value	*q*‐value	Heterogeneity test Method	*Q*	*p*	Pleiotropy test P intercept	F
*Allisonella*	8	IVW	1.42(1.18‐1.70)	0.0002	0.0095	MR Egger	7.5303	0.2746	0.5220	141.7226
		MR Egger	2.21(0.60‐8.12)	0.2767	0.9962	IVW	8.1101	0.3230		
		Weighted median	1.36(1.06‐1.74)	0.0140	0.8358					
		Weighted mode	1.41(1.01‐1.95)	0.0814	0.9927					
*Bacteroides*	8	IVW	2.25(1.48‐3.42)	0.0001	0.0095	MR Egger	7.8403	0.2500	0.8000	25.6915
		MR Egger	1.64(0.15‐17.71)	0.6964	0.9962	IVW	7.9319	0.3386		
		Weighted median	1.77(1.02‐3.07)	0.0408	0.9660					
		Weighted mode	1.53(0.76‐3.12)	0.2720	0.9927					
*Candidatus_Soleaferrea*	9	IVW	0.75 (0.60‐0.95)	0.0149	0.3896	MR Egger	2.5464	0.9236	0.7951	69.0352
		MR Egger	1.04(0.97‐11.19)	0.9740	0.9962	IVW	2.6191	0.9560		
		Weighted median	0.81(0.60‐1.09)	0.1582	0.9660					
		Weighted mode	0.84(0.54‐1.31)	0.4644	0.9927					
*Eubacterium_coprostanoligenes*_group	13	IVW	0.67(0.47‐0.95)	0.0230	0.5013	MR Egger	13.9713	0.2346	0.7524	23.3107
		MR Egger	0.83(0.20‐3.40)	0.8028	0.9962	IVW	14.1042	0.2941		
		Weighted median	0.71(0.45‐1.12)	0.1375	0.9660					
		Weighted mode	0.69(0.34‐1.41)	0.3278	0.9927					
*Fusicatenibacter*	18	IVW	0.71(0.52‐0.97)	0.02897	0.5422	MR Egger	20.5265	0.1974	0.6537	23.4805
		MR Egger	0.55(0.17‐1.76)	0.3275	0.9962	IVW	20.7946	0.2356		
		Weighted median	0.63(0.43‐0.93)	0.0191	0.8358					
		Weighted mode	0.56(0.31‐1.03)	0.0810	0.9927					
*Lactobacillus*	8	IVW	0.72 (0.58‐0.90)	0.0046	0.1491	MR Egger	2.4210	0.8772	0.2740	81.2322
		MR Egger	1.00(0.56‐1.78)	0.9962	0.9962	IVW	3.8703	0.7946		
		Weighted median	0.78(0.58‐1.05)	0.1001	0.9660					
		Weighted mode	0.81(0.56‐1.17)	0.2950	0.9927					
Ruminococcus_torques_group	7	IVW	0.41 (0.25‐0.65)	0.0002	0.0095	MR Egger	3.1094	0.6831	0.2465	20.7688
		MR Egger	1.57(0.20‐12.45)	0.6886	0.9962	IVW	4.8313	0.5656		
		Weighted median	0.40(0.21‐0.76)	0.0050	0.6511					
		Weighted mode	0.39(0.16‐0.91)	0.0728	0.9927					

**FIGURE 2:**
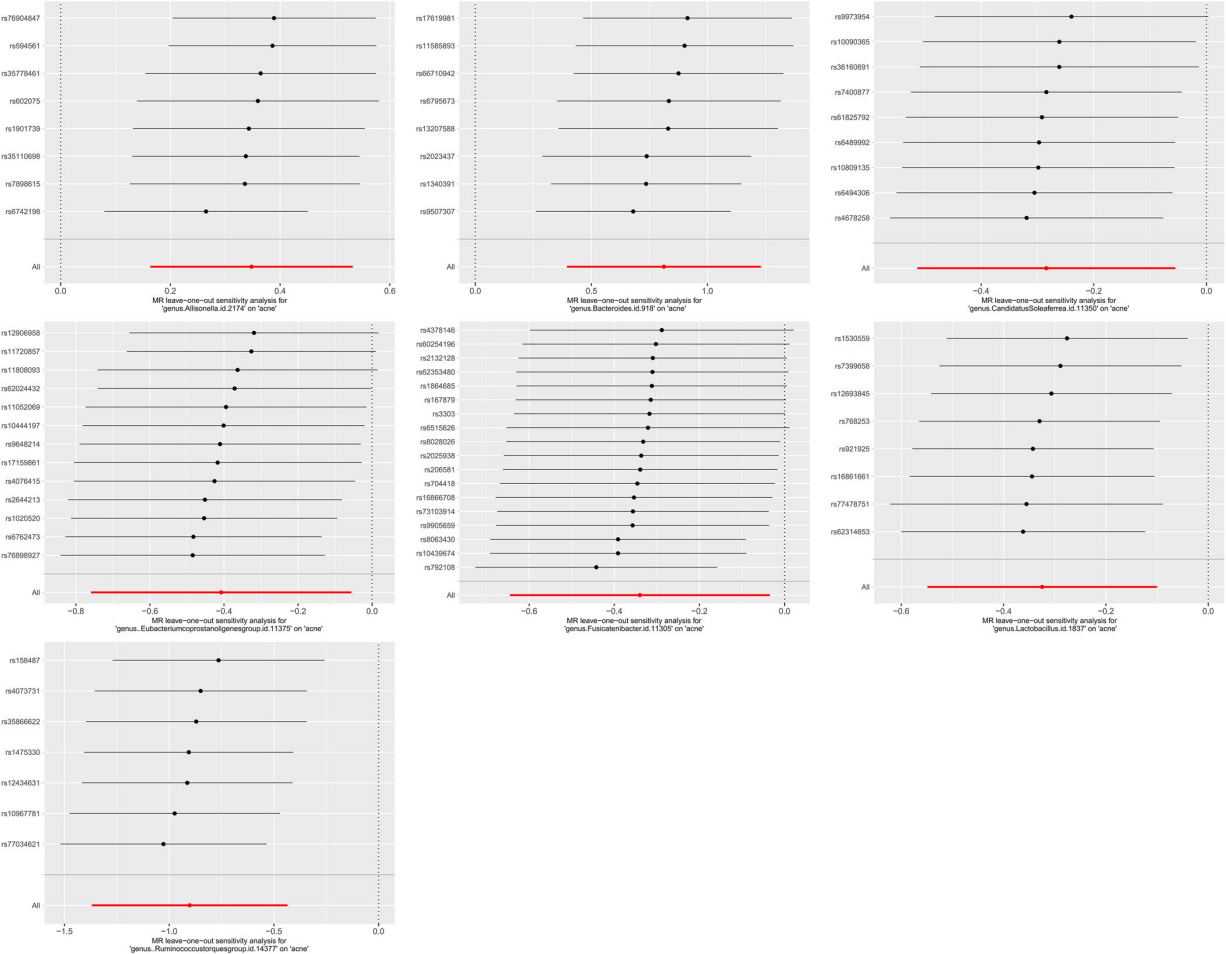
Leave‐one‐out plots for the causal association between gut microbiota and acne.

Among the 71 causal associations evaluated, the minimum and maximum F‐statistics for the instrumental variables (IVs) were 14 and 206, respectively, indicating an absence of weak instrument bias. Heterogeneity and pleiotropy results are presented in Table 1, and no statistical significance (*p* > 0.05) was found. The leave‐one‐out method, illustrated in Figure 2, confirmed the stability and reliability of the causal effect estimates.

## 4 DISCUSSION

The present study sought to assess the causal relationship of gut microbiota and acne through a two‐sample MR analysis. Data from the FinnGen project and the MiBioGen consortium was used. The findings indicated that *Ruminococcus torques* group was protective against acne. Moreover, four other gut microbiota genera, including *Candidatus soleaferrea* and *Eubacterium coprostanoligenes* group, showed a suggestive protective effect against acne. Conversely, the presence of *Allisonella* and *Bacteroides* was found to aggravate acne.

Two groups of gut microbiotas, *Bacteroides* and *Lactobacillus*, have been clinically investigated for their association with acne. One of the most numerous species in the human gut flora, *Bacteroides*, are essential to various biological processes. Studies have suggested that *Bacteroides* may contribute to acne development by degrading polysaccharides and may enhance the inflammatory response, stimulate hyperangiogenesis, and weaken immune defenses.^32^ A study conducted on acne grading and microbiology found that the abundance of *Bacteroides* was notably higher in individuals with grade 4 acne compared to those with grades 1–3. This observation suggests that the excessive growth of *Bacteroides* may contribute to the worsening of acne.^33^ *Bacteroides*, as a Gram‐negative bacterium, has a pathogenic site mainly in membrane vesicles, the secretion of which is essential for bacterial physiology and pathogenesis.^34^ *Lactobacillus*, a popular probiotic in vivo, is beneficial for improving skin health.^35^, ^36^ Specifically, according to several reports from Korea, kimchi intake brings *Lactobacillus* plantarum strains to the body, and these strains can improve acne vulgaris, exerting a wide range of anti‐inflammatory and anti‐pathogenic bacterial activities that effectively regulate bacterial flora in the skin.^37^, ^38^ In addition, the Koreans found that tea tree oil fermented in *Lactobacillus* improved acne better than regular tea tree oil, reducing inflammatory markers and insulin‐like growth factor 1 receptor.^39^ Moreover, a Chinese study on gut microbes in patients with moderate and severe acne vulgaris observed reduced *Lactobacillus* levels,^12^ supporting the findings of this study.

The human intestinal flora undergoes a series of metabolic processes, culminate in the production of SCFAs such as acetic acid, butyric acid, and propionic acid.^40^, ^41^ SCFAs play a crucial role in reducing inflammation in patients with acne. They inhibit the activation of Toll‐like receptor‐2 (TLR‐2) by *Propionibacterium* acnes by inhibiting HDACs, which reduces the release of inflammatory substances like IL‐6, IL‐8, and TNF‐α.^42^ Additionally, SCFAs can lower the intracellular pH of *propionibacterium* acnes, leading to decreased survival of the bacterium, which ultimately improves acne symptoms.^43^ In the present study, it was observed that *Candidatus soleaferrea*, one of the microflora, is capable of producing SCFAs and secreting GLP‐2, which is a crucial nutritional hormone responsible for keeping the function and structure of the intestinal epithelium.^44^ The anaerobic bacterium, *Eubacterium coprostanoligenes* group, has been found to have the ability to lower cholesterol levels in the body. Moreover, it can produce beneficial SCFAs and has the potential to improve dyslipidemia.^45^, ^46^, ^47^ In a study of fecal transplanted intestinal flora and functional constipation, *Fusicatenibacter* was found to produce butyric acid and valeric acid, decreasing IL‐8 expression.^48^ In colorectal patients, *Lactobacillus* was found to secrete SCFAs to enhance the intestinal barrier and has anti‐inflammatory action.^49^ *Lactobacillus rhamnosus* GG (LGG) was found to increase *Ruminococcus torques* group abundance in weaned piglets and further promote the production of SCFAs.^50^ Based on the aforementioned evidence, it can be inferred that there exists a close correlation between the aforementioned microflorae and SCFAs, which could be one of the underlying reasons for their capacity to ameliorate acne.

According to the widely accepted theory of the gut‐skin axis, the mechanism by which the gut microbiota influences acne may be associated with the inflammatory immune response.^51^ The abundance of *Allisonella* is significantly increased in patients with Down syndrome and is positively associated with the levels of proinflammatory cytokines.^52^ According to a reported study, *Bacteroides* were found to stimulate macrophages and monocytes to secrete the proinflammatory cytokine TNFα through an LPS‐mediated pathway.^53^ Medium‐chain triglycerides (MCT) improve the immune status by increasing the abundance of *Fusicatenibacter* in the intestinal flora.^54^ Moreover, mice with colitis observed a suppression of colitis‐related responses in the IL‐23/Th17 axis in vivo and a reduction in the secretion of proinflammatory cytokines after oral administration of *Lactobacillus*.^55^ *Lactobacillus* maintains immune homeostasis through T regulatory (Treg) cells,^56^ and the role of individual gut flora in the inflammatory immune response may influence the progression of acne.

While the present study has several strengths, including the minimization of confounding factors and using sensitivity analysis to ensure result reliability, it also has some limitations. Firstly, since the study used summary statistics instead of raw data, it was not possible to perform subgroup analysis or investigate nonlinear relationships. Secondly, the study was conducted at the genus level, and investigating the correlation between gut microbiota and acne at the specie level was not feasible. To meet the standards of sensitivity analysis and horizontal pleiotropy testing, the significance threshold was established at *p* <1.0 × 10^−5^, enabling the inclusion of additional genetic variation as an instrumental variable. However, this approach also introduced SNPs that may violate the MR assumptions. For this reason, an FDR correction was performed. The study focused on European populations, limiting the generalizability of the findings to other ethnic populations. Future research should include high‐quality GWASs of diverse ethnic groups to address these limitations. Moreover, it is important to note that MR is an epidemiological tool and further experimental studies are necessary to explore the mechanisms by which gut microbiota contributes to the pathogenesis of acne.

## 5 CONCLUSION

To summarize, the current two‐sample MR study provides evidence supporting a causal link between gut microbiota and acne. The seven identified gut microbiota (*Allisonella, Bacteroides, Candidatus soleaferrea*, *Eubacterium coprostanoligenes* group, *Fusicatenibacter, Lactobacillus, Ruminococcus torques* group) may provide new insights into the prevention and treatment of acne.

## CONFLICT OF INTEREST STATEMENT

The authors state that the research has no commercial or financial associations that could give rise to perceived conflict of interest.

## ETHICS STATEMENT

Ethical review and approval were not sought for this study involving human participants, as it adhered to all relevant local legislation and institutional guidelines. Written informed consent from the participants' legal guardians or next of kin was not necessary, as it aligned with national legislation and institutional requirements.

## Supporting information

Supporting Information

Supporting Information

## Data Availability

Datasets analyzed in this study can be found in MiBioGen repository at https://mibiogen.gcc.rug.nl/ ^20^ and the FinnGen repository at https://r8.finngen.fi/.^21^
